# Gene Banks Pay Big Dividends to Agriculture, the Environment, and Human Welfare

**DOI:** 10.1371/journal.pbio.0060148

**Published:** 2008-06-10

**Authors:** R. C Johnson

## Abstract

Plants are the foundation of human life. The Western Regional Plant Introduction Station is a vital link in a greater US and international system of gene banks providing plant genetic resources to improve agriculture and the environment.

Nearly a century after the pioneering American apple tree purveyor Johnny Appleseed traveled from town to town planting nurseries in the Midwestern United States, Frans Nicholas Meijer left his Netherlands home to pursue a similar vocation as an “agricultural explorer” for the US Department of Agriculture. Over the course of his career, Meijer, who changed his name to Frank Meyer after reaching the New World, helped introduce over 2,500 foreign plants from Europe, Russia, and China, including the lemon that would bear his name. Starting with his first expedition for Asian plants in 1905, Meyer would encounter isolation, physical discomfort, disease, robbers, and revolutionaries in his quest to collect useful plants.

Although some of Meyer's collections are still used today, only a few are conserved in their original form. They, along with countless other collections from the early 20th century, disappeared because there was no long-term system for conservation. To rectify this problem, Congress established a system of repositories after World War II to maintain and distribute plant genetic resources. This resource has now grown into the National Plant Germplasm System (NPGS; http://www.csrees.usda.gov/nea/plants/in_focus/pbgg_if_npgs.html) and consists of 26 repositories with approximately half a million individual collections. The NPGS functions to maintain agricultural biodiversity and ensure the preservation of the genetic resources needed for food security and environmental restoration.

## Reducing Genetic Vulnerability with Germplasm

As crop genetic resources continue to erode worldwide, the need to acquire and maintain germplasm is ongoing and urgent. Since agriculture's beginnings about 10,000 years ago, approximately 7,000 of Earth's 300,000 plant species have been used as crops. The Food and Agriculture Organization estimates that today only 150 plant species are under extensive global cultivation, with 12 crop species providing 80% of the world's food. Although modern agriculture feeds more people on less land than ever before, it also results in high genetic uniformity by planting large areas of the same species with genetically similar cultivars, making entire crops highly vulnerable to disease, drought, and insect infestation. The Irish potato famine of the mid-1800s, which claimed an estimated 1.5 million lives, and the heavy losses to the 1970 US corn crop from southern corn leaf blight, costing an estimated US$1 billion, provide dramatic illustrations of the risks of genetic uniformity and dependence on just a few crops.

According to the World Conservation Union, one in every eight plant species in the world, and one in three in the US, is threatened with extinction, resulting largely from the development of rural land and invasion of alien species. Many of these threatened plant species may harbor unknown attributes that could benefit agriculture or the environment. With climate change, some plant populations may disappear completely. Expanding conservation to preserve seeds and genetic resources through gene banks has become critically important, yet it is only one component of a larger conservation plan aimed at preserving plants in situ on farms, in reserves, and in their native habitats. Though the challenge is great, there is more effort and interest than ever.

**Figure 1:**
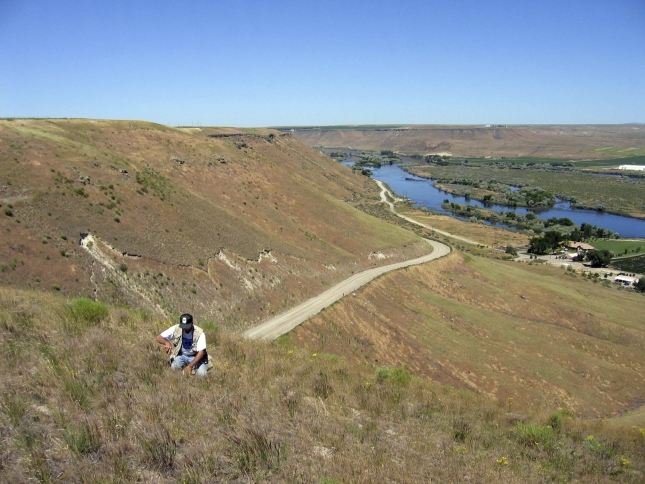
Collecting Accessions Walter Kaiser collects taper-tip onion (Allium acuminatum) along the Snake River in Idaho in 2005. Collections were made across a broad area of Western rangeland to strengthen the WRPIS Allium collection and for research to determine taper-tip onion adaptation zones needed for successful revegetation.

**Figure 2:**
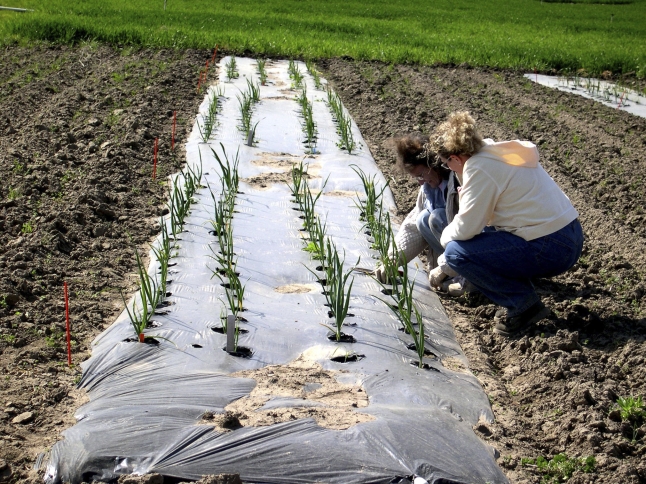
Regenerating Seeds Accessions must be regenerated when the quality or quantity of seed is low. Here Marie Pavelka and Terri LeClaire (foreground) plant leek accessions for seed regeneration. Each plot will be covered with a screen cage to prevent crossing with other accessions, and bluebottle flies will be introduced into the cages as pollinators.

As part of the NPGS, the Western Regional Plant Introduction Station (WRPIS; http://www.ars.usda.gov/main/site_main.htm?modecode=53481500) in Pullman, Washington, acquires, maintains, conserves, evaluates, and distributes plant genetic resources needed to improve crops, develop new crops, and provide plant material for environmental restoration. Accessions (distinct, uniquely identified samples of seeds or plants maintained as part of a germplasm collection) are acquired through field exploration and exchanges with other gene banks and organizations (see Figure 1). To ensure that collections are free of invasive weeds and pests, accessions entering the US are inspected by the Animal and Plant Health Inspection Service. Seeds from new collections are cleaned, their taxonomy verified, tested for viability, and annotated with population and ecological information from collection locations. These “passport data” are available to all on the Germplasm Resources Information Network (http://www.ars-grin.gov/npgs/). When the quantity or quality of seeds is low, an increase or grow-out is needed to make seeds available for distribution (see Figure 2). This is done using growth chambers, greenhouses, and field facilities under strict guidelines to preserve the genetic integrity of accessions. Each year, more than 1,600 accessions and 100,000 plants are grown for seed increase at the WRPIS.

**Figure 3:**
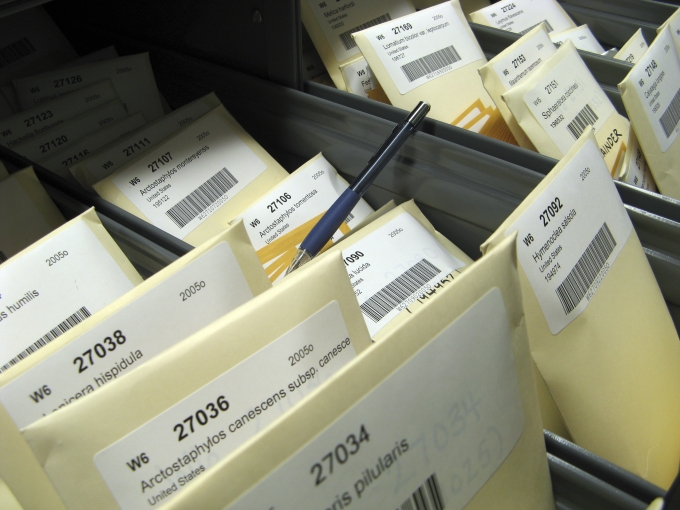
Storing Native Seeds Packages of native North American accessions in cold storage at the WRPIS. The WRPIS maintains approximately 4,500 accessions of native species, more than 1,500 of which have been acquired in the last five years as part of a cooperative project with the Bureau of Land Management's Seeds of Success Program (http://www.nps.gov/plants/sos/).

The WRPIS collection of approximately 80,000 accessions is an “active collection” maintained under intermediate-term storage at 4 °C to facilitate management and distribution (see Figure 3). Long-term, security back-up of accessions is provided at –18 °C or in liquid nitrogen at the National Center for Genetic Resources Preservation (NCGPR; http://www.ars.usda.gov/Main/docs.htm?docid=8071) in Fort Collins, Colorado. Each year the WRPIS fills approximately 1,500 seed orders, sending out about 20,000 seed packets to researchers worldwide. Much of the screening and development for disease, insect, and abiotic stress resistance is done through the germplasm user community; that is, those researchers requesting germplasm, but scientists at the WRPIS also do this work, often in cooperation with off-station users. The grasses are the largest collection with more than 18,000 accessions and 107 genera. Other major collections include beans (more than 15,000 accessions with 50 taxa represented), alfalfa (more than 7,500 accessions with 78 taxa), peas (more than 4,000 accessions with 12 taxa), and beets (more than 2,500 accessions with 16 taxa).

## Mining Gene Banks for Novel Attributes

Plant breeders are still finding useful genes within these collections, and plant explorers are still uncovering specimens from diverse regions and climates with new attributes, paving the way for the creation of innovative agricultural tools. Useful germplasm is “where one finds it,” and screening procedures range from traditional field evaluation trails to advanced DNA marker technology. Yet the time between collection and utilization is typically years or even decades. When the major sugar beet disease rhizomania was found in North America in the 1980s, resistance depended on a single gene, *Rz1*. Understanding that single gene resistance would be vulnerable to new virus strains, plant breeders developed other sources of resistance. These included resistance in a sugar beet from Turkey collected in 1952 and in an accession from Denmark collected in 1985, both in the WRPIS collection. By 2002, the rhizomania virus had overcome the *Rz1* resistance, and these new forms of resistance are now being used as the next line of defense against the disease. This type of “race for genes,” the need to stay ahead of new and variant forms of pests, relies on the continual supply of germplasm from gene banks and new collections.

Recently, a new type of insect-resistance for grasses was developed using WRPIS collections acquired from Morocco in the 1990s. These collections were examined for fungal endophytes, which live inside plants and produce alkaloids that can be toxic to grazing animals and insects. In this mutually beneficial living arrangement, the fungus receives a protected living environment and the plant receives resistance to most types of herbivory. Some endophytes in the Morocco collections were found to produce alkaloids that are benign to livestock but still repel insects. This endophyte has been incorporated into modern grass varieties, providing a novel form of pesticide insect resistance.

As important as these foreign introductions are to protecting food security, preserving native genetic resources is critical to the conservation and restoration of natural environments. Much of the Western US is dominated by rangelands that support grazing, wildlife, and recreation. In the summer of 2007, wildfires, located mostly in the West, burned an estimated 14,000 square miles at a cost of US$1.8 billion. Although fire is a natural part of the rejuvenation cycle, exotic weeds are crowding out natives and providing increased fuel supply for rangeland fires. Downy brome, first introduced to North America in the mid-1800s from the Mediterranean region of Europe, is one of the most pervasive and recalcitrant weeds on Western rangelands, and together with drought and global warming has increased the frequency and severity of fires, degrading habitat for livestock and wildlife. In cooperation with the Bureau of Land Management, the WRPIS is collecting key native species for revegetation and restoration efforts to help restore the natural balance between habitat and fire. To accomplish this restoration, the WRPIS is working to determine linkages between adaptive genetic variation and climatic variables across the landscape. This will ensure that germplasm chosen for revegetation will be adapted to local environments, and as such, more resilient and sustainable. Given the complexity of the ecological interactions disrupted by wildfires, no single approach offers all the answers, but any effective strategy will surely rely on plant genetic resources.

## Providing a Link to Global Food Security

In the last 40 years, genetic conservation efforts have grown nationally and internationally. Established in 1971, the Consultative Group on International Agricultural Research (http://www.cgiar.org/) supports a system of 11 International Agricultural Research Centers with substantive germplasm collections. These collections are held in trust on behalf of humanity, and are available without restriction. There is a longstanding spirit of cooperation between the International Centers and the NPGS gene banks. Recently, a project to improve chickpea production has involved the WRPIS and the International Crops Research Institute for the Semi-Arid Tropics (ICRISAT; http://www.icrisat.org/) in India. Chickpeas are the third most important food legume globally, and more than 95% of their production and consumption is in developing countries. Wild chickpea species from both the WRPIS and ICRISAT collections are being used to find new germplasm with resistance to pod-boring insects. The goal is to transfer the resistance in wild species to domestic chickpeas, and thus improve food production.

In 1992, the Convention on Biological Diversity called on the international community to sign a global agreement aimed at covering all aspects of biological diversity, including biodiversity conservation, sustainable use, and fair and equitable sharing of benefits arising from genetic resources. So far, 189 countries have signed on, including the United States.

On another front, the Svalbard Global Seed Vault (http://www.nordgen.org/sgsv/), located within the Arctic Circle on the Norwegian island of Spitsbergen, recently opened with the mission of guarding against loss of germplasm in traditional gene banks from natural or political disasters throughout the world. Designed to withstand threats from global warming, the underground vault, dug deep in the permafrost, can safely store up to 1.5 billion seeds representing 3 million varieties. Even though the NCGPR has long provided security backup for the US, no true global facility existed before Svalbard. The need for these facilities is well illustrated by the example of teff, a cereal grain from Ethiopia used to make injera, a gluten-free pancake-like bread. Teff is a staple in Ethiopia, and international interest in teff for food and forage has grown dramatically in recent years. During the Ethiopian civil war in the 1980s, the national gene bank was ransacked and much of the germplasm lost, including teff. As the Ethiopian gene bank was revitalized in the late 1980s, other gene banks helped restore Ethiopian collections. As part of this process, the WRPIS repatriated 336 accessions of Ethiopian teff collected by the US in the 1960s. The Svalbard Global Seed Vault gives all countries a secure backup facility that includes full ownership rights.

There is little doubt that plant genetic resources are needed to address today's problems. Yet the erosion of plant biodiversity continues a decline that had already begun even as Frank Meyer traveled the globe in search of botanical resources to benefit society. Seed bank collections serve as insurance against unanticipated future threats to food security, the degradation of our environment, and the loss of plant biodiversity. The WRPIS, as part of the US network of plant gene banks, provides a vital link in an emerging world system aimed at maintaining, conserving, and utilizing these precious plant genetic resources, the seeds of our future.

## Abbreviations

ICRISAT: International Crops Research Institute for Semi-Arid Tropics
NCGPR: National Center for Genetic Resources Preservation
NPGS: National Plants Germplasm System
WRPIS: Western Regional Plant Introduction Station

